# Phase Separation in Ge-Rich GeSbTe at Different Length Scales: Melt-Quenched Bulk versus Annealed Thin Films

**DOI:** 10.3390/nano12101717

**Published:** 2022-05-18

**Authors:** Daniel Tadesse Yimam, A. J. T. Van Der Ree, Omar Abou El Kheir, Jamo Momand, Majid Ahmadi, George Palasantzas, Marco Bernasconi, Bart J. Kooi

**Affiliations:** 1 Zernike Institute for Advanced Materials, University of Groningen, Nijenborgh 4, 9747 AG Groningen, The Netherlands; d.t.yimam@rug.nl (D.T.Y.); a.j.t.van.der.ree@rug.nl (A.J.T.V.D.R.); j.momand@gmail.com (J.M.); majid.ahmadi@rug.nl (M.A.); g.palasantzas@rug.nl (G.P.); 2 Department of Materials Science, University of Milano-Bicocca, Via R. Cozzi 55, I-20125 Milano, Italy; o.abouelkheir@campus.unimib.it (O.A.E.K.); marco.bernasconi@unimib.it (M.B.)

**Keywords:** phase change materials, Ge-rich GST, pulsed laser deposition, phase separation, GGST, EDX elemental chemical mapping, embedded memory, density functional theory

## Abstract

Integration of the prototypical GeSbTe (GST) ternary alloys, especially on the GeTe-Sb_2_Te_3_ tie-line, into non-volatile memory and nanophotonic devices is a relatively mature field of study. Nevertheless, the search for the next best active material with outstanding properties is still ongoing. This search is relatively crucial for embedded memory applications where the crystallization temperature of the active material has to be higher to surpass the soldering threshold. Increasing the Ge content in the GST alloys seems promising due to the associated higher crystallization temperatures. However, homogeneous Ge-rich GST in the as-deposited condition is thermodynamically unstable, and phase separation upon annealing is unavoidable. This phase separation reduces endurance and is detrimental in fully integrating the alloys into active memory devices. This work investigated the phase separation of Ge-rich GST alloys, specifically Ge_5_Sb_2_Te_3_ or GST523, into multiple (meta)stable phases at different length scales in melt-quenched bulk and annealed thin film. Electron microscopy-based techniques were used in our work for chemical mapping and elemental composition analysis to show the formation of multiple phases. Our results show the formation of alloys such as GST213 and GST324 in all length scales. Furthermore, the alloy compositions and the observed phase separation pathways agree to a large extent with theoretical results from density functional theory calculations.

## 1. Introduction

Phase change materials have been heavily investigated for many applications in fields of phase-change memories (PCMs), nanophononics, and neuromorphic applications for the past few years [1,2]. Although there have been many promising binary and ternary alloys with phase switching properties, by far the most studied and integrated phase change materials are the Ge-Sb-Te alloys, specifically Ge_2_Sb_2_Te_5_ or GST225. Despite being the center of research in the field with attractive crystallization properties and stability and being the front runner up for future memory and optoelectronic devices, GST225 is by no means without its limitations. One main reliability issue, especially crucial for PCMs, is the gradual “drift” of resistance in the metastable amorphous phase of GST225 over time [3,4,5]. The resistivity drift towards higher values, due to structural relaxations to a thermodynamically stable state [6], is particularly detrimental when going beyond two memory states per bit, e.g., for neuromorphic computing. Other issues, such as void formation and electromigration, have also been reported [7,8]. In addition, the low crystallization temperature of GST225 (≈150 °C) limits the material’s potential for automotive and aeronautics applications [9]. Simply put, GST225 could not meet the automotive specifications and soldering threshold needed in embedded memory applications [10,11].

There has been a growing trend in recent years in shifting away from pure GST225 alloy for PCMs to find a better alternative with high crystallization temperature and thermal stability. One way of achieving the needed properties is by “doping” pure GST225 with impurities [12,13]. Incorporating impurities into GST225 induced higher crystallization temperature, better structural and thermal stability, and faster switching [8,14]. Interestingly, among all doping impurities, nitrogen doping is by far the most attractive and results in promising experimental works in the field [15,16,17,18,19,20]. Another approach is to deviate in composition from the stable GST225 phase by incorporating excess Ge, creating Ge-rich GST alloys. Increasing the Ge content in the GST alloys shows an increase in the crystallization temperature, promoting high data retention and endurance [10,21,22]. Moreover, in addition to Ge enriching, nitrogen doping of Ge-rich GST alloys has also been studied for increased thermal stability and higher crystallization temperatures, which are attractive for future memory devices [9,23,24].

A common similarity in almost all Ge-rich GST alloys is thermodynamically unfavorable initial phases. Although research on the alloys produced many promising results, phase separation into stable phases and local compositional variation upon melt-quenching could not be avoided [9,25,26,27]. This issue poses a significant problem for programming operations in an active device. The local composition variation and phase separation into Ge-rich and Sb-rich regions compromise the device’s functionality [10,22,28,29]. Moreover, the excess Ge content in Ge-rich alloys increases oxidation susceptibility with lowered crystallization temperature and Te enrichment [9,30]. This work reports experimental results of local phase separation in a Ge-GST alloy GST523. The choice of the specific starting composition, i.e., GST523, is due to previous experimental and theoretical results on Ge-rich GST alloys on the Ge-Sb_2_Te_3_ tie-line [31,32]. Among a series of Ge-rich GST, GST523 shows a high crystallization temperature, while the Ge content is still moderately low. Therefore, the alloy’s phase separation into GSTxyz and pure Ge is still limited. In addition, GST523 is an interesting alloy to investigate, since it is on the Ge-Sb_2_Te_3_ tie-line and can also be potentially produced by alternating thin Ge and Sb_2_Te_3_ layers. We provide evidence for the formation of multiple phases upon melting and quenching of a thermodynamically unstable Ge-rich GST phase at different length scales. A large area morphology and elemental composition of a Ge-rich GST sample were analyzed using scanning electron microscopy (SEM). In addition, we use scanning/transmission electron microscopy (S/TEM) for composition analysis of pulsed laser deposited Ge-rich GST alloys on a smaller length scale. The experimental results have been compared with calculations based on Density Functional Theory (DFT) on the thermodynamics of the decomposition pathways.

## 2. Experimental Methods

We prepared thin films of Ge-rich GST, GST225, and Sb_2_Te_3_ using pulsed laser deposition (PLD), with a KrF excimer laser operating at 248 nm wavelength. For GST225 and Sb_2_Te_3_ depositions, powder-sintered targets from K-TECH were used. For the deposition of Ge-rich GST thin films, an in-house target was made. Exact constituents of high-purity Ge, Sb, and Te atomic portions were sealed in a vacuum quartz ampule. The ampule was put into an oven and melted by gradually increasing the temperature to 950 °C and kept for 2 h. Finally, the ampule was cooled by water and crushed into a powder material. The ingot was ball milled for 5 h to produce a fine powder, which was then used to produce pellets of 20 mm diameter by a pressure press. Finally, the pellets were sintered at 300 °C for 1.5 h to produce a dense powder target with a composition of GST523. A fluence of 0.8 J cm^−2^, processing gas (Ar) pressure of 0.12 mbar, and target-substrate distance of 55 mm were used for all depositions. The depositions were performed at room temperature to produce as-deposited amorphous samples. Reflection high-energy electron diffraction (RHEED) was used as an initial characterization for the amorphous nature of deposited thin films. Thin films were deposited on Si wafer covered with thermal SiO_2_ film for ellipsometry analysis and on continuous carbon and Si_3_N_4_ TEM grids for scanning/transmission electron microscopy (S/TEM) imaging.

For scanning electron microscopy (SEM), a FEI Helios G4 CX and a FEI NovaNanoSEM 650 (both from Thermo Fisher – FEI, Hillsboro, OR, USA) equipped with an energy dispersive X-ray (EDX) detector were used to analyze the surface morphology of the initial crystalline ampule and the final powder-sintered target used for deposition. A Themis Z S/TEM (Thermo Fisher – FEI, Hillsboro, OR, USA) operating at 300 kV and a JEOL 2010 TEM (JEOL USA Inc., Boston, MA, USA) operating at 200 kV were used for plan-view imaging and elemental analysis of deposited thin films. A cross-sectional specimen of the as-deposited and crystalline Ge-rich GST thin films was prepared with a focused ion beam (FIB) (FEI Helios G4 CX). 

Dynamic ellipsometry measurements (DE) were conducted to investigate the phase transformations of the as-deposited amorphous thin films. A heating stage (HTC-100), attached to a J. Woollam UV-VIS variable angle spectroscopic ellipsometer (VASE), was used for the measurements. The ramp and hold steps of the measurements were controlled by the TempRampVASE software (Version 1.06), while the WVASE software (Version 3.916) monitored the measurement intensities. All DE measurements were conducted in air at a 70° incidence angle and with a 5 °C min^−1^ heating rate. Spectroscopic ellipsometry (SE) measurements were performed on the thin films before and after heating. Measurements were conducted in the spectral range of 300–1700 nm. For fitting ease, maximum accuracy, and reduction of parameter correlation, measurements at three angles of incidence (65°, 70°, and 75°) were collected. For refractive index (n) and extinction coefficient (k) extraction, measurement data were fitted with the Tauc–Lorentz oscillator model using the WVASE fitting software.

## 3. Results and Discussion

**Figure 1:**
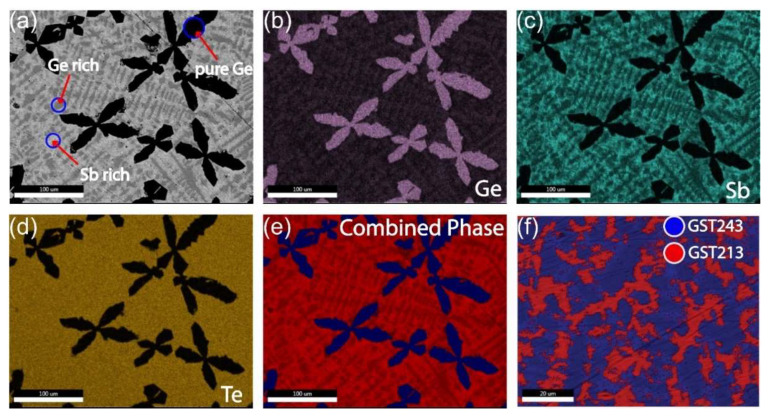
SEM–EDX mapping of water-quenched crystalline alloy showing the formation of multiple phases. (**a**) SEM image showing the formation of Ge dendrites from the excess germanium in the system and other phases. (**b**) Ge, (**c**) Sb, and (**d**) Te elemental maps. (**e**) A combined (Ge, Sb, and Te) elemental mapping with clear color contrast shows three phases. (**f**) When taken away from the pure Ge dendrites, an elemental map shows two distinct phases. The scale bar in (**a**–**e**) is 100 µm and in (**f**) is 20 µm.

To investigate the phase separation of a relatively large liquid volume of Ge-rich GST alloy, we produced a GST523 ingot heated to 950 °C for a sufficiently long time to ensure that a completely homogeneous GT523 liquid is obtained. The quartz tube is rapidly quenched into water by pulling it out of the furnace directly into a water bath at room temperature. The strong driving force for phase separation of GST523 combined with the relatively fast cooling produces phase separation on the scale of 1–100 μm which can be very well assessed by SEM and also EDX performed in the SEM. Figure 1a shows a backscatter image with Z contrast and, therefore, the observed dark phase is Ge-rich and the observed brighter phase in the image is Sb-rich. The EDX results in Figure 1b–e show that primary Ge dendrites are formed. The remaining liquid solidifies with relatively homogeneous Te concentration but with complementary Ge and Sb concentrations, i.e., phase separation in regions (1) high in Ge and low in Sb and (2) low in Ge and high in Sb. In extreme form, these two phases could be GeTe and Sb_2_Te_3_, respectively, but EDX shows that this is incorrect. Thus, the observed morphology and EDX results are also consistent; i.e., (pure) Ge forms the largest primary dendrites, because it is the phase with the highest melting temperature that solidifies first; then the Ge-rich GST phase solidifies as smaller secondary dendrites, because it has an intermediate melting temperature; and, finally, the remaining Sb-rich GST solidifies last, since it has the lowest melting temperature. The morphology of the phases observed in SEM (also higher magnification images than shown here), the large scale on which phase separation has occurred, and the well-known fact that GST alloys are very poor glass formers make it inevitable that all three distinct regions are crystalline in nature.

Detailed quantitative analysis of SEM-EDX maps shows that the following three “phases” are formed: Ge, GST213, and GST243. Here, the term “phases” is associated with the local compositional variations observed from the experimental results. It also holds for the STEM-EDX results presented below. However, keep in mind that the local compositions can describe a real stable crystalline phase, such as pure Ge, but also metastable phases observed outside the GeTe-Sb_2_Te_3_ tie-line. In case of water-quenching a relatively bulky liquid sample present in evacuated quartz tube, the cooling rates are relatively low, and the crystalline phases formed are similar to the trigonal stable GST phases observed in ref. [33]. Nevertheless, in this respect, GST213 can be considered with GeTe as starting phase, where about one third of the Ge is replaced by Sb, and GST243 as a Ge-rich and Te-poor GST with excess Sb. The overall composition for the GST523 sample measured for the EDX map shown in Figure 1e gave on average (in at.%) 47.1 Ge, 21.8 Sb, and 31.1 Te (with quantification error in at.% of 2.7 for Ge, 1.4 for Sb, and 1.4 for Te), which is reasonably close to the intended one. Additional information on the SEM–EDX analysis is presented in Supplementary Materials Section S1. The observed deviation from GST523 probably originates from the coarseness of the primary Ge dendrites. Then, even for a large area, such as that shown in Figure 1a, considerable fluctuations can occur depending on how much of the Ge dendrites are exactly in the analyzed area. Since we know the overall composition, which is GST523, and we know reasonably the composition of the three phase-separated phases (Ge, GST213, and GST243), we can estimate the fraction of atoms present in each of the three phases. Then 30% of the atoms are in Ge, 40% in GST213, and 30% in GST243. When, for simplicity, assuming that the atomic densities are identical in the three phases (which of course is a relatively crude approximation), then the same three fractions can hold for the volume fraction of the three phases in the material and, when sufficiently randomly distributed, also the area fraction in Figure 1a. When looking at Figure 1a qualitatively, these fractions appear quite reasonable. However, the EDX mapping provides quantitative output regarding the area fraction of the different phases. For example, Figure 1f quantifies the fractions of the GST213 (secondary dendritic phase) and GST243 as 60% and 40%, respectively, which matches well the 4:3 ratio estimated above. 

**Figure 2:**
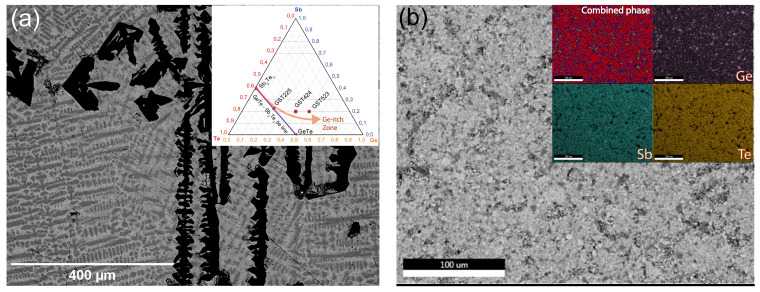
(**a**) GST523 crystalline target made by melting a combination of constituent elements at 950 °C. Clear phase separation is visible from the image. Inset shows a phase diagram of Ge-Sb-Te alloys indicating the tie-line of Sb_2_Te_3_-GeTe and the deviation toward Ge-rich GST regions. (**b**) Powder-sintered target, prepared by crushing the crystalline target for better yield and stoichiometric transfer. Inset shows SEM EDX mapping of the powder-sintered target. A “single phase”-like powder target of GST523 was produced.

In PLD, crystalline and amorphous (dense) targets can be used to ablate materials with a high-power laser and create thin-film layers on a given substrate. In another way, and relatively common in PLD, powdered sintered (porous) targets can be used as target materials for ablation. Combining individual elements of the necessary components and milling them into a fine powder, which is then pressed into a pellet and sintered in a furnace, will produce a usable target material. Of course, when dealing with the accurate weight of individual elements, there is always a risk of deviating from the correct final composition due to the loss of elements in the ball milling and mixing processes. Therefore, in the present work, multiple routes have been investigated to produce Ge-rich GST thin films. Since Ge-rich GST alloys can be considered Ge addition to Sb_2_Te_3_, GST523 can be seen as (Ge)5 + (Sb_2_Te_3_)1. Based on this, we produced “superlattice-like” heterostructures with alternating pure Ge and Sb_2_Te_3_ layers. The details of Ge target production and heterostructure depositions are explained in Supplementary Materials Section S1. Although the desired composition was achieved by varying the individual layer thicknesses, the produced films showed severe problems with Ge oxidation and delamination (see Supplementary Materials Section S2 (Figures S1 and S2). Another alternative was to initiate the deposition process directly from the crystalline target. Along the GeTe-Sb_2_Te_3_ tie-line, Ge_x_Sb_y_Te_z_ ternary alloys have multiple known stable phases, with minimum or no phase separation (see Figure 2a inset). However, when Ge content is increased and deviates away from this tie-line, the stability of the alloy is thermodynamically unfavorable, and phase separation is inevitable.

Figure 2a shows a backscatter SEM image of a (dense) crystalline GST523 PLD target with clearly distinguishable phases present. The laser spot size for most PLD set-ups is much larger (3–9 mm^2^) than the segregated individual grains of 10–100 µm range. Therefore, we could assume some form of homogeneity inside the laser–target interaction volume during ablation. However, in our deposition using the crystalline GST523 target depicted in Figure 2a, the thin-film composition deviated significantly from the initial target stoichiometry. One primary reason is that the ablation threshold and characteristics are different for individual phases, with different elemental compositions present in the target material. As a next alternative, we crushed the crystalline ingot into a fine powder, then compacted it into a pellet using high pressure. The pellet was sintered at an elevated temperature in the final step. Figure 2b shows an SEM image of the final powder-sintered target, and the large area SEM-EDX analysis shows the homogeneous distribution of the elemental constituents in the inset. Although still with an overall GST523 composition, we created in this way a “pseudo-single phase” powder target for deposition. Thin films of Ge-rich GST were deposited, and the stoichiometry transfer and the film quality were dramatically improved when going from the initial crystalline target (Figure 2a) to the powder-sintered target (Figure 2b).

Once a suitable target was made for material ablation, multiple depositions were performed to create amorphous as-deposited Ge-rich GST samples. Depending on the technique used, the as-deposited phase of PCMs have different structures and properties. It is especially crucial when considering the thermal stability of the produced material after melt-quenching. Therefore, it is ideal to start with an as-deposited phase that closely resembles the melt-quenched phase structure. Although attractive for large-scale production with usually a homogeneous chemical composition in the as-deposited phases, sputtering techniques often produce samples with reduced thermal stability after melt-quenching [34]. On the other hand, pulsed laser deposited samples should have structures already more closely resembling phases after melt-quenching. One piece of evidence for this is the presence of nanocrystals embedded in the amorphous matrix of pulsed laser deposited thin films (especially Sb_2_Te_3_). However, it is not desirable to have a crystalline phase inside the amorphous matrix initially at room temperature deposition, but this also means that the as-deposited phase will not change dramatically after the melt-quench process, which is also known to produce crystalline embryos embedded in an amorphous matrix [35,36].

**Figure 3:**
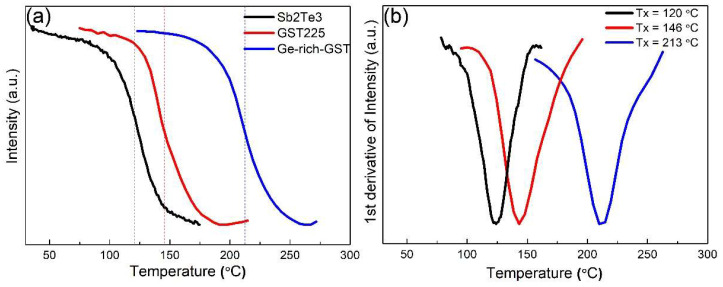
(**a**) Crystallization curves from dynamic ellipsometry measurements. Measurements of Sb_2_Te_3_ and GST225 are also plotted, in addition to the Ge-rich GST thin film, for better comparison. An abrupt change in intensity indicates a phase transformation upon heating. (**b**) The first derivative of the intensity from the dynamic ellipsometry measurement indicates exact crystallization temperature. A higher Tx value is observed for Ge-rich GST samples.

The variation of optical properties between amorphous and crystalline phases in phase change materials can be probed continuously to monitor the phase transition upon heating. Reflectance data are continuously monitored in dynamic ellipsometry (DE) measurements while the sample is heated with a constant temperature ramping. We have also prepared thin films of Sb_2_Te_3_ and GST225 for DE measurements for better insight into crystallization temperature variations with Ge content. Spectroscopic ellipsometry measurements and data fittings are also given in Supplementary Materials Section S4 (Figure S4). Figure 3a shows the results from DE measurement for Sb_2_Te_3_, GST225, and Ge-rich GST thin films. All films have thicknesses of 35–40 nm, and a heating rate of 5 °C min^−1^ was used. The normalized intensity shows the measured value for the ellipsometry parameter ψ at 1400 nm wavelength. The figure shows abrupt changes in the measured parameter upon phase transformation for all three phases. In Figure 3b, the first derivatives of the normalized measurement values are given. By fitting the curves with a Gaussian function, we can accurately extract the crystallization temperature for the thin films. Sb_2_Te_3_ and GST225 films crystallize at 120 °C and 146 °C, respectively. However, for Ge-rich GST thin films, crystallization happens at a higher temperature of 213 °C. Our Ge-rich GST thin films, thus, show Tx values that are about 70 °C higher than that of GST225 thin films. Given the excess Ge content present in the Ge-rich GST thin films, the relatively higher Tx is not surprising. It has been shown that increasing Ge content leads to higher Tx values for the ternary phase [10,31].

Crystallization dynamics studies on PCM thin films, with and without capping layers, showed the effect of oxidation on the crystallization onset. In general, uncapped films show transition temperatures lower than capped thin films. The reduction in the phase transition temperature is associated with heterogeneous nucleation at the oxidized regions of the uncapped thin films, which has been shown in crystallization dynamics works on prototypical PCMs such as GST225 and GeTe [37,38,39]. The effect worsens for the Ge-rich GST thin films containing excess elemental Ge with high oxidation affinity. The Ge migration towards the top surface (as evident in Figure 5a,c) contributes heavily to the phase separation, creating Ge-depleted regions in the lower parts of the thin film. The Ge depleted regions might have a transition temperature lower than other phases present in the thin film. It is worth mentioning here that we have to be cautious when using the term “crystallization temperature” for Ge-rich GST alloys. Given the complexity of the crystallization dynamics and the phase separations in the samples, it might be wise to use the term “transition temperature”.

**Figure 4:**
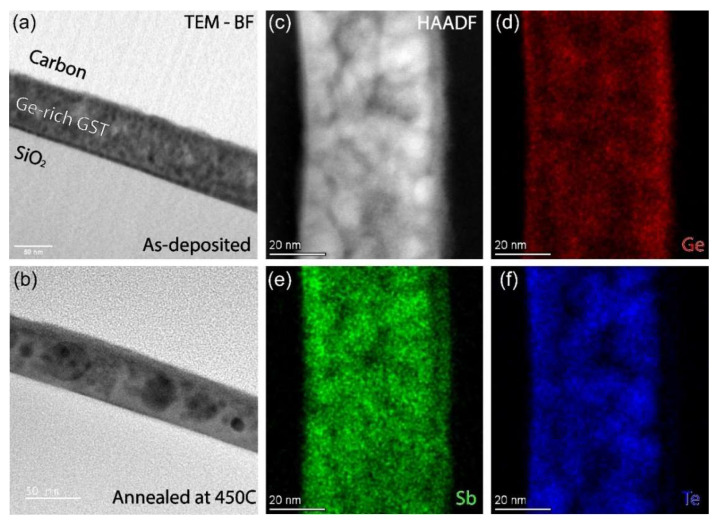
BF-TEM images of Ge-rich GST thin film in (**a**) as-deposited phase and (**b**) after annealing at 450 °C for 30 min. High-angle annular dark-field (HAADF)-STEM image (**c**) and STEM-EDX chemical compositions of (**d**) Ge, (**e**) Sb, and (**f**) Te for the annealed Ge-rich GST sample.

TEM-EDX analysis of the as-deposited Ge-rich GST thin films shows an average composition (in at.% and with atomic error <2% for all elements) of 46 Ge, 22 Sb, and 32 Te, which deviates from the initial target composition GST523. This deviation is caused by the formation of Ge-rich particulates (see Supplementary Materials Section S3 (Figure S3). Formation of particulates is inherent to most PLD systems. Figure 4 shows cross-sectional images and EDX results of as-deposited and annealed Ge-rich GST samples obtained using TEM and S/TEM characterization. Figure 4a,b show BF-TEM images of a Ge-rich GST thin film before crystallization and after annealing at 450 °C for 30 min. Brighter and darker areas are visible in the as-deposited phase, and the image contrast is not homogeneous. The contrast is attributed to the local variation in composition and not because of diffraction contrast [26]. After annealing, the local composition is disturbed due to clusters of grain formations with different compositions. The first reasonable assumption for the image contrast would be that, since Sb and Te have comparable atomic numbers, Ge-rich and Ge-poor regions are present. This assumption is then verified by the high-angle annular dark-field (HAADF)-STEM images and STEM-EDX chemical mappings. Figure 4c shows the HAADF image for the annealed Ge-rich GST sample with clear contrast with local composition variations. Figure 4d–f shows the chemical mapping distribution of Ge, Sb, and Te, respectively. Comparing the HAADF image with the EDX mappings, it is clear that the darker regions in the image indeed represent Ge-rich zones. Although some local variation in all constituents is present, the Sb composition shows a relatively homogeneous map compared to Ge and Te. The homogeneous Sb intensity is contradictory to the results found in the SEM-EDX analysis of the melt-quench crystalline target, where Te was relatively homogeneous. This discrepancy could be attributed to the length scale associated with the measurement areas. The measurement and analysis are in the nm range for the thin film and the µm range for the crystalline target. Underlying is that the length scales in the crystalline targets are associated with quenching relatively slowly from the melt at high temperature, whereas the length scales in the thin film are associated with heating the solid (initially amorphous) phase. Of course, decomposition can occur much faster over long distances in liquid at high temperature than in solid at low temperature.

**Figure 5:**
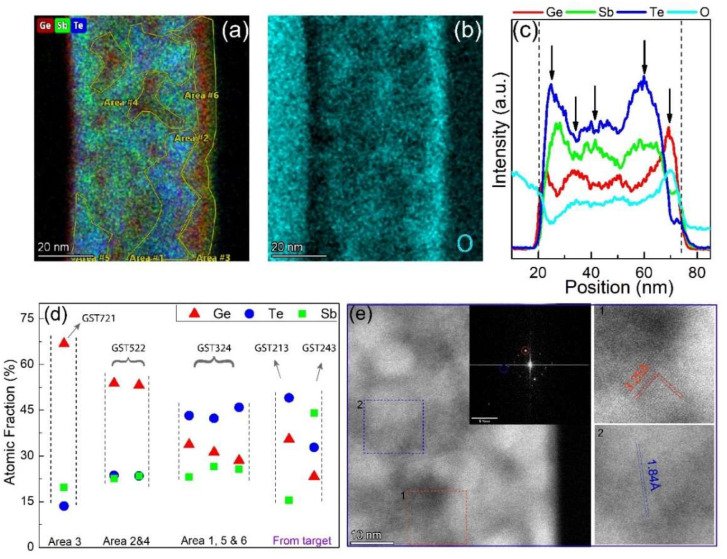
(**a**) The combined (Ge, Sb, and Te) STEM-EDX chemical mapping of the Ge-rich GST sample. Based on color contrast, multiple areas are selected for local composition analysis. The EDX chemical composition of oxygen is given in (**b**). (**c**) The line profile of the combined and the oxygen STEM-EDX chemical mapping of the Ge-rich GST sample. The oxygen line profile is scaled for better comparison with the Ge line profile. (**d**) Atomic fraction (in at.%) of the individual selected areas in (**a**) are plotted and compared with the atomic fraction from the melted target. (**e**) High-resolution images of some grains in the annealed sample with the inset showing the FFT.

To further investigate the phase decomposition and formation of multiple phases in the Ge-rich GST thin films and compare the STEM-EDX results with the SEM-EDX analysis of the crystalline target, we need to analyze the local composition variation in the thin film. A somewhat crude way of investigating the local chemical variations is by analyzing the color contrast in the combined chemical mapping, in addition to the intensity line profiles. The combined chemical mapping and the selected areas for local composition analysis are given in Figure 5a. The line intensities of all elements (presented in Figure 5c) change throughout the film thickness, indicating the presence of multiple regions with different compositions and phase separations. The phase separations are associated with the Ge-rich phases and Te/Sb-rich regions. As can be seen from the line profiles, an increase in Ge intensity is accompanied by a decrease in Te/Sb intensity and vice versa. What is interesting is the migration of Ge to the film surface. This Ge migration is strongly driven by oxidation, which pulls Ge atoms to the surface, and this is well observable in cross-sections of thin films such as in Figure 5, but we also observed this for GST225 nanoparticles, where after prolonged exposure to air at room temperature the particles with an initial homogeneous composition develop a clear Ge-oxide outer shell [40]. However, also below the top surface the Ge-rich areas appear oxidized, as there is a clear direct correlation between Ge and oxygen concentration (see Figure 5b,c). However, it is important to note that this oxidation occurs when the very thin TEM lamella produced by FIB is some time in air before it is inserted in the TEM. Therefore, this subsurface oxidation did not affect the phase separation process during the thermal anneal. Figure 5e shows a high-resolution STEM image of some of the grains found in the sample. Most of the grains in the image appear to be relatively pure Ge crystals (with d-spacing of 3.25 Å). Another grain (with a lower d-spacing of 1.84 Å) is also presented which could not be indexed to any known Ge or GST phase.

A closer look at Figure 5a shows the presence of three primary color variations that stand out. This color variation is highly dependent on Ge content. What that means is that we can interpret the colors as high Ge content or “deep red” (near the film surface and represented by area #3), medium Ge content or “light red” (area #2 and #4), and low Ge content or “no red” (area #1, #5, and #6). Multiple areas from the different color groups have been selected, and their local composition is plotted in Figure 5d. For comparison, we also plotted the actual compositions (in at.%) of GST213 and GST243 phases identified and measured in the melt-quenched crystalline target. Note that the pure Ge phase found in the crystalline target is not included in the plot. Results from similar color groups have been averaged, and a composition for the tertiary alloy is extracted. As expected, the three regions show three different local compositions of GST721, GST522, and GST324. We can find multiple similarities and differences between the STEM-EDX and SEM-EDX results based on the plot in Figure 5d. The first and obvious similarity is the formation of pure Ge (close to pure for GST721) phase in the Ge-rich GST thin film and the melted crystalline target. The pure Ge presence is not surprising, since the initial composition has excess Ge. The SEM image in Figure 1a and the high-resolution STEM image in Figure 5e both confirm the presence of pure Ge. Another similarity is the presence of a Te-rich phase in both samples. GST324 in the annealed thin film and GST213 have comparable properties in the plot such that they both are higher in Te, similar in Ge, and lower in Sb compositions. One could even approximate the GST324 alloy as GST213 + GeTe.

Despite the similarities, some glaring differences exist between the compositions extracted from the thin film and from the crystalline target. The first is a Sb-rich phase in the crystalline target (GST243) which was not found in the thin film. Another difference is the phase GST522 found in the thin film, closely resembling the initial GST523 phase. It is worth noting that the difference in some of the local compositions of the thin film from the crystalline target could be attributed to multiple factors. One reason could be the difference in mobility of the constituents in the thin film and the melted target due to the supplied heat to the system. Starting from the melt, at 950 °C, would have the upper hand in providing enough energy to the system compared with annealing at 450 °C for 30 min. In addition, atomic mobilities are much higher in the liquid than in the solid. We could even argue that the GST522 phase is the prime example of the slow diffusion of constituents in the solid thin film, since it is close to the starting GST523 composition. Another factor for the difference could be the length scale used for the measurement. For the thin film, due to the high accelerating voltage of the S/TEM (300 kV compared to the 30 kV for SEM), the resolution in the chemical mapping is much better. Thus, the S/TEM-EDX provides an opportunity to correctly probe the local composition of the sample within nm scales. In the SEM-EDX, however, individual pixel sizes were limited to µm length scale for our sample. Therefore, the order of magnitude difference in resolution could produce aggregated/averaged compositions of nm length scale in a single µm length pixel, thus providing a biased estimation.

Nevertheless, it is expected that both techniques capture the correct length scale, since it is inherently longer by quenching, rather slowly (despite using water quenching), a bulk material from liquid at high temperature compared to heating a solid thin film at relatively low temperature. In a true memory device, of course, melt-quenching is also employed, but then, using short electrical pulses (<100 ns) and very small material volumes that are switched, melt-quenching can be extremely fast, thereby limiting phase separation. Nevertheless, Ge-rich GST alloys are very susceptible to phase separation, and even in such memory devices it is unlikely that it can be suppressed completely.

**Figure 6:**
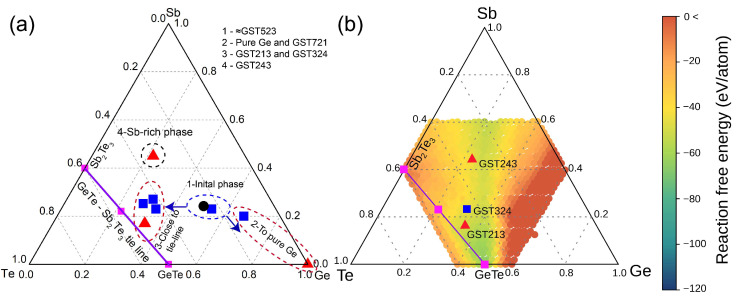
(**a**) The phase diagram of the starting composition and the final phases found from the STEM-EDX and SEM-EDX results. The black circle represents the initial phase of the thin film ((in at.%) 46 Ge, 22 Sb, and 32 Te). The blue squares represent elemental compositions extracted from STEM-EDX analysis of the annealed thin film. Finally, the red triangles show the compositions of phases found from the melt-quenched crystalline target in SEM-EDX analysis. The GeTe-Sb_2_Te_3_ tie-line, with Sb_2_Te_3_, GeTe, and GST225 compositions, are also presented for comparison. (**b**) Map of the decomposition pathways during crystallization of GST523 as based on DFT calculations (see ref. [27]). The color code gives the reaction free energy (meV/atom) to form the cubic alloys on the ternary phase diagram starting from the GST523 reactant. The same color code of the decomposition maps in ref. [27] for alloys on the Ge-GST124 tie-line is used.

The phase separation of Ge-rich GST alloys has been theoretically investigated by means of DFT calculations to identify possible separation pathways [27,32,41]. To identify the similarities and differences between our result and that of the theoretically predicted phase separations, we plot the results from STEM-EDX and SEM-EDX analyses in the ternary phase diagram as presented in Figure 6a. The plot identifies four phases: 1—the starting phase, 2—the pure Ge phase, 3—phases close to the GeTe-Sb_2_Te_3_ tie-line, and, finally, 4—a Sb-rich phase found in the large scale SEM-EDX analysis of the melt-quenched crystalline target. 

When comparing our experimental results with previous DFT results for the GST523 alloy [32], both agree that Ge must be formed. Our experimental results do not show alloy formation on the GeTe-Sb_2_Te_3_ tie-line nor the formation of Sb_2_Te_3_, meaning that the phases seen are more likely metastable cubic and not trigonal. The DFT work shows that the formation of metastable cubic phases such as GST221 and GST323 became competitive when moving away from the trigonal phases. This observation seems to align with our experimental results, given the compositional similarities (GST323 from simulation to GST324 of STEM-EDX and GST213 of SEM-EDX results) and the energetically favorable decomposition into two phases (e.g., Ge and GST234) instead of three phases (e.g., Ge, GeTe, and GST221). Indeed, one apparent deviation from the theoretical results of ref. [32] is the presence of Sb-rich GST phase GST243 in our experimental result. We must, however, consider that only a subset of the possible decomposition pathways was analyzed in ref. [32]. In addition, Bordas et al. [42] provided an extensive analysis of the Ge-GeTe-Sb_2_Te_3_-Sb phase diagram, allowing the prediction of solidification paths and solidification sequences. For alloys in a very large region around the Ge-Sb_2_Te_3_ tie-line, the formation of pure Ge is inevitable during solidification. It is also predicted that forming a ternary phase close to pure Ge is highly unlikely, which we also report in our results. However, a major difference with the predictions of Bordas et al. occurs regarding the formed GSTxyz phases in addition to pure Ge. During solidification of alloys on the Ge-Sb_2_Te_3_ tie-line, the solidification steps according to Bordas et al. always contain alloys only found on the GeTe-Sb_2_Te_3_ tie-line. When cooling from high temperature they reported the formation of three alloys, GST225, GST124, and GST147, in different composition (and temperature) ranges. This deviates from our observation when cooling from the melt of GST523 alloy, where we observed the formation of GST213 and GST324 clearly deviating from the GeTe-Sb_2_Te_3_ tie-line. Nevertheless, the solidification sequence of, firstly, Ge and then, secondly, GST213 (resembling GeTe) and, thirdly and finally, GST243 (rather close to ‘δ-Sb_2_Te’), as deduced from the results related to Figure 1, is very much in line with the predictions of Bordas et al.

To improve over the previous theoretical analysis, we have here adopted the more systematic approach exploited in our previous work [27] to study the decomposition of alloys on the Ge-GST124 tie-line. The DFT formation free energy of the alloys in the metastable cubic phase was provided in ref. [27] for all compositions in the central part of the ternary phase diagram. The calculated free energy consists of the total energy at zero temperature and the configurational free energy (at room temperature) due to disorder in the cubic sublattices. We used this information to compute the reaction free energy for the decomposition pathways of GST523 into a generic Ge_x_Sb_y_Te_z_ cubic alloy plus elemental Ge, Sb, and Te and the binary GeTe and Sb_2_Te_3_ compounds, as discussed in ref. [27], to which we refer for all details. This calculation yields the map of the decomposition free energies shown in Figure 6b. Each point in the map gives the value of the decomposition free energy for the formation of the corresponding alloy from GST523. The vibrational contribution to the reaction free energy, not included in Figure 6b, was shown to be negligible [32]. The more negative the reaction free energy is, the more favored is the corresponding decomposition channel. The green regions in Figure 6b, thus, correspond to the more favorable decomposition products which do include the three alloys GST213, GST324, and GST243 seen experimentally.

The decomposition map in Figure 6b indicates that the formation of alloys close to GeTe should be thermodynamically favored as well. However, as discussed in ref. [32], the formation of alloys close to GeTe requires a strong segregation of both elemental Ge and Sb which might be kinetically more difficult. Kinetic hindrances might, thus, explain the absence of alloys close to the GeTe composition in the experimental samples.

## 4. Conclusions

In summary, chemical mappings and local phase separations at different length scales have been investigated in Ge-rich GST alloys using SEM-EDX and STEM-EDX techniques. For a bulk crystalline sample, water-quenching produced three thermodynamically favored phases. These phases are pure Ge, Ge-rich phase, and Sb-rich phases. In addition to the SEM-EDX measurements on a relatively large length scale, STEM-EDX analyses have been performed on a smaller length scale on annealed Ge-rich GST thin films. For deposition of initial Ge-rich GST as-deposited thin films, pulsed laser deposition parameters and target preparation steps were optimized. Dynamic ellipsometry measurements on the as-deposited Ge-rich GST thin films have been used to determine the crystallization temperatures accurately. Pulsed laser deposited Ge-rich GST thin films have an elemental composition close to GST523, and their crystallization temperature was 70 °C higher than holds for the prototypical GST225 alloys. STEM-EDX analysis on the annealed Ge-rich GST thin films shows that thermal treatment at 450 °C for 30 min induced phase separation into pure Ge and Ge-poor GST phases. One would assume that, because of thermodynamics, the phases observed after separation would lie on the GeTe-Sb_2_Te_3_ tie-line. Interestingly, that is not the case here. The identified phases actually lie in between the GeTe-Sb_2_Te_3_ and the Ge-Sb_2_Te_3_ tie-lines. This outcome was predicted in a previous theoretical work [32] to be due to the formation of the metastable cubic phases instead of the trigonal ones. Our DFT calculation of the reaction free energy provides a comprehensive map for the decomposition pathways of GST523 which encompass the compositions seen experimentally among those thermodynamically favored. Our work provides experimental and theoretical evidence to the possible separation pathways in Ge-rich GST alloys, which is a valuable input in choosing future Ge-rich GST alloys for phase change memory applications. 

## Data Availability

Data can be available upon request from the authors.

## Supplementary Materials

The following supporting information can be downloaded at: https://www.mdpi.com/article/10.3390/nano12101717/s1, Figure S1: Transmission electron microscope image of a thin film produced by alternating sublayers of Ge and Sb_2_Te_3_. The number of pulses used to ablate the Ge and Sb_2_Te_3_ targets was relatively small to approximate the deposition as a “co-sputtering”. The idea of creating an intermixed layer in the as-deposited amorphous phase was not successful, since the film was not stable, and the electron beam caused delamination. Figure S2: (a) High-angle annular dark-field (HAADF)-STEM image of a thin film cross-section produced by alternating Ge and Sb_2_Te_3_ layers. In (b–e) EDX mappings of the elements Ge, Sb, Te, and O are presented. (f) The line profiles of the different elements analyzed by STEM-EDX chemical mapping of the heterostructure. The oxygen line profile follows the Ge line profile, indicating the formation of GeO_x_ layer. Figure S3: (a) A large area view of the Ge-rich GST thin film surface. Multiple particulates can be seen in all windows of the TEM grid. (b) A zoomed-in image of particulates in one of the windows. (c) An amorphous as-deposited layer of the Ge-rich thin film is produced when moving away from the particulates. Figure S4: Optical constant data extracted from spectroscopic ellipsometry measurements and data fitting using Tauc–Lorentz optical oscillator. (a) Index of refraction for amorphous and (b) crystalline samples of Sb_2_Te_3_, GST225, and GST523 thin films. (c) The extinction coefficient for amorphous and (d) crystalline samples of Sb_2_Te_3_, GST225, GST523 thin films.
