# Supplementary material for: Phase Separation in Ge-Rich GeSbTe at Different Length Scales: Melt-Quenched Bulk versus Annealed Thin Films
Source: Nanomaterials (Basel). 2022 May 18;12(10):1717. doi: 10.3390/nano12101717 (PMC9143932; doi:10.3390/nano12101717)
Supplement: Supplementary file 1 [file nanomaterials-12-01717-s001.zip › nanomaterials-1712138-supplementary.pdf]

SI 1 – SEM–EDX Analysis.

Figure 1 of the main text shows results of the SEM-EDX analysis of the melt-quenched bulk sample, and the conclusion is drawn there that the following three 'phases' are formed: Ge, GST213, and GST243. However, it is good to know for the GST compounds what exact compositions have been measured using EDX and how these have been simplified to the discrete GST phase. Note that we know that three distinct phases are formed based on the distinct morphologies observable in the SEM images. Then, for the phase we identify as GST213, the actual composition measured (in at.%) was on average 35.5 Ge, 15.5 Sb, and 49.1 Te in one area and 33.5 Ge, 18.2 Sb, and 48.3 Te in another area. Including the typical error bars in this composition analysis of 2 to 3 at.%, it is a reasonable step to simplify this morphological phase to GST213. Similarly, for GST243 the actual composition measured (in at.%) was on average 23.3. Ge, 44.0 Sb, and 32.8 Te in one area and 21.9 Ge, 47.1 Sb, and 31.0 Te in another area.

SI 2 – Ge-Sb_2Te_3 alternating layers.

One approach used to create a Ge-rich GST thin film was to alternate Ge and Sb_2Te_3 depositions. For this deposition, to combine with the commercially available Sb_2Te_3 powder target, a homemade Ge target was made. Large Ge crystals were ball milled into fine powder to produce a powder target. Then, similar to the Ge-rich GST target preparation steps (described in the experimental methods section of the main text), the fine powder is pressed into circular pellets, which were then sintered in an oven. For this approach, two different deposition ways have been investigated. The first is to ablate Ge and Sb_2Te_3 layers alternatively but in a somewhat "co-sputtering" approach. The idea is to ablate a small number of constituents from each target to intermix in the as-deposited sample. A recipe script was used to automate the alternative deposition with specific pulse numbers and repetition rates. Thirty alternation steps were carried out to produce the thin film with 20 pulses of Sb_2Te_3 and 200 pulses of Ge for individual steps. A repetition rate of 1 Hz and 10 Hz was used for Sb_2Te_3 and Ge targets, respectively. After the final step, a capping layer of LaAlO3 was deposited to prevent oxidation. Although it was possible to produce a thin film in this approach, it was unstable. Fig. S1 shows the plan view TEM image of the deposited thin film. It is clear to see that the electron beam was enough to induce delamination, fracture and roll-up, probably because the film was under stress initially during deposition.

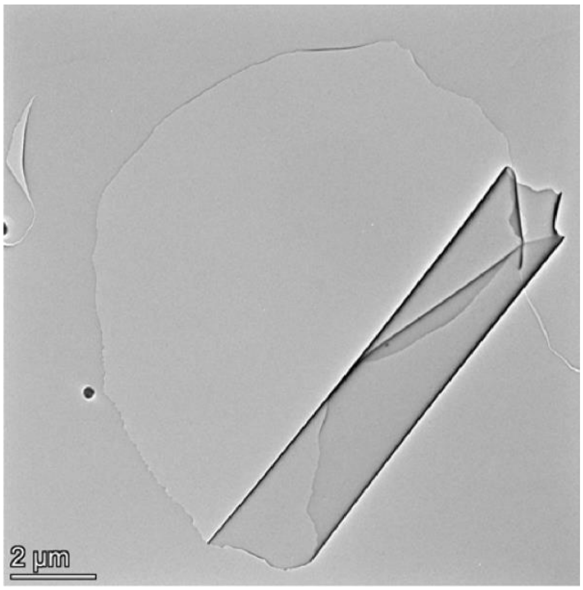

Figure S1. Transmission electron microscope image of a thin film produced by alternating sublayers of Ge and Sb_2Te_3. The number of pulses used to ablate the Ge and Sb_2Te_3 targets was relatively small to approximate the deposition as a “co-sputtering.” The idea of creating an intermixed layer in the as-deposited amorphous phase was not successful since the film was not stable, and the electron beam caused delamination.

In the second step, we have moved away from ‘intermixing’ at the as-deposition amorphous phase and moved to truly alternating layers. Here the individual layers are relatively thick (Sb_2Te_3 layers of 8 nm each and Ge layers of 10 and 15 nm). The produced thin-film heterostructure is visible from STEM imaging and EDX elemental mapping of the cross-section in Fig. S2. The image clearly shows individual layers of Sb_2Te_3 and Ge, with an interface between them. The overall composition of the heterostructure turned out close to the desired value of GST523 since we can control the individual layer thicknesses to achieve the overall composition. However, there is a serious oxidation problem, as is evident from the chemical mapping. The oxidation of the Ge target mainly causes this during preparation. Overall, the idea of alternatively depositing Ge and Sb_2Te_3 layers seems promising at first. The capability of achieving any Ge-rich GST alloy by varying layer thickness is desirable. However, the formation of unstable as-deposited phase and severe oxidation of the Ge layer during PLD hinders our progress.

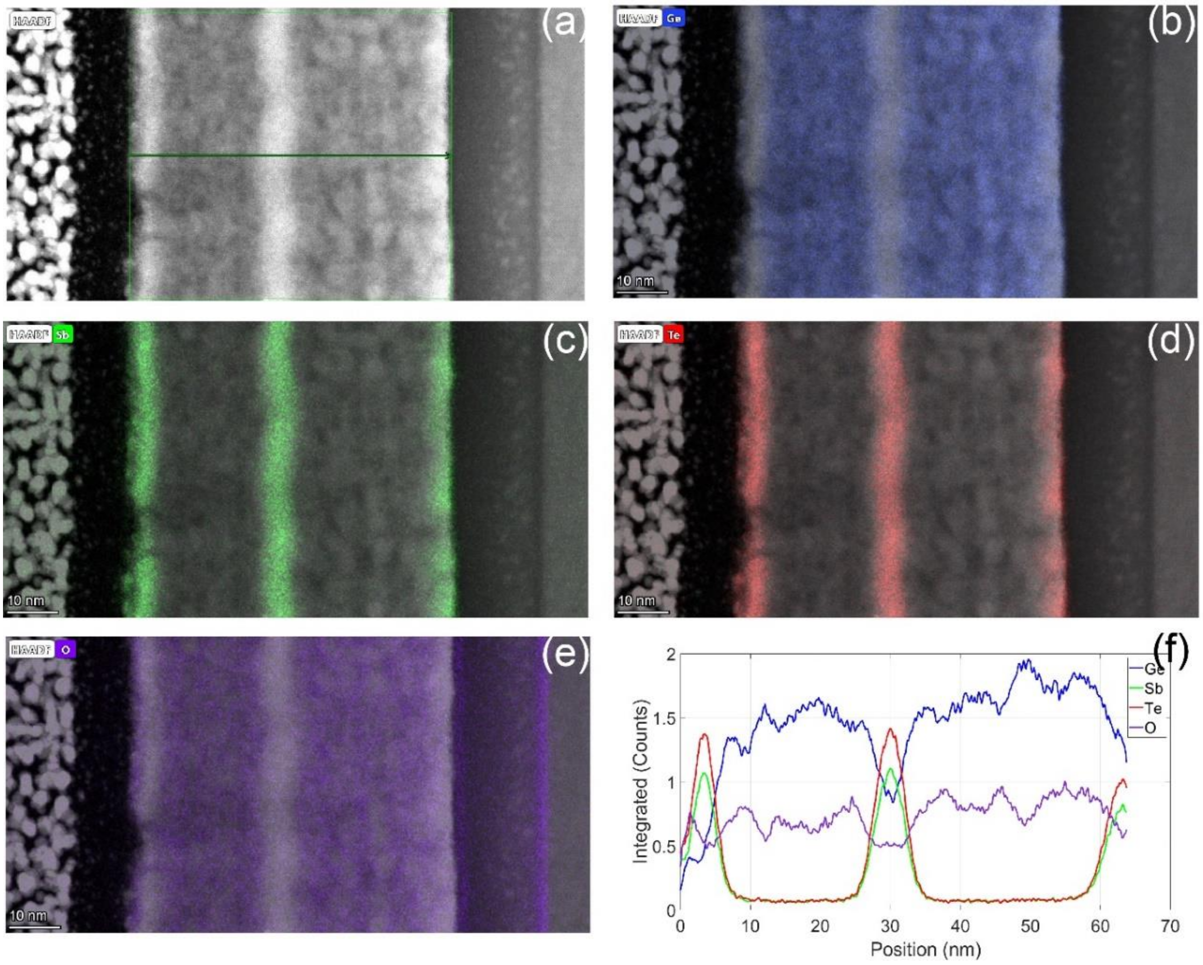

Figure S2. (a) High-angle annular dark-field (HAADF)-STEM image of a thin film cross-section produced by alternating Ge and Sb_2Te_3 layers. In (**b**), (**c**), (**d**), and (**e**) EDX mappings of the elements Ge, Sb, Te, and O are presented. (**f**) The line profiles of the different elements analyzed by STEM-EDX chemical mapping of the heterostructure. The oxygen line profile follows the Ge line profile, indicating the formation of GeO_x layer.

SI 3 – Ge-rich GST thin film depositions and particulates.

After a suitable target was prepared for ablation, Ge-rich GST thin films were deposited at room temperature in the as-deposited amorphous phase. When comparing the final composition of the produced thin films with the initial target, there is a slight reduction in the Ge content. The formation of Ge-rich particulates causes this reduction. The particulate formation is relatively common in PLD. Fig. S3 shows the plan-view TEM images of a Ge-rich GST thin film surface. The particulates are visible in the images.

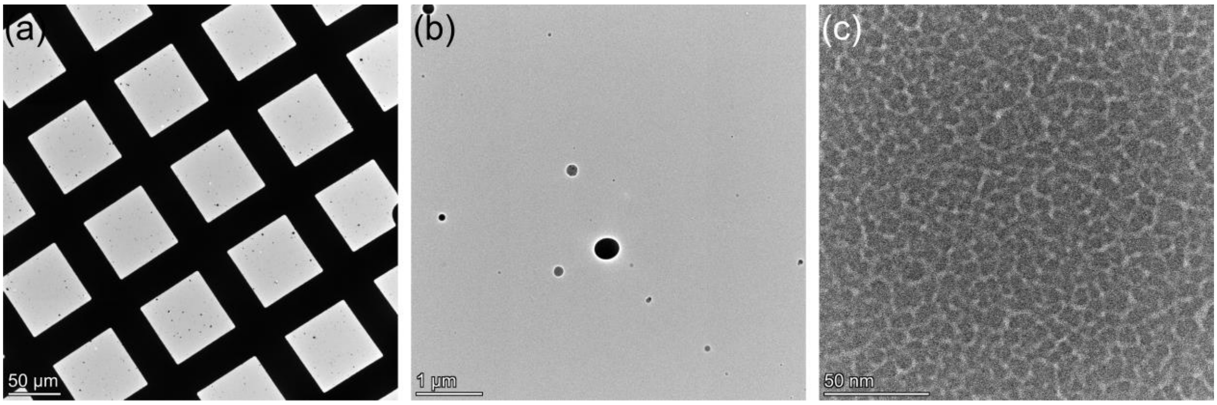

Figure S3. (**a**) A large area view of the Ge-rich GST thin film surface. Multiple particulates can be seen in all windows of the TEM grid. (**b**) A zoomed-in image of particulates in one of the windows. (**c**) An amorphous as-deposited layer of the Ge-rich thin film is produced when moving away from the particulates.

SI 4 Spectroscopic ellipsometry measurements and data fittings

Before and after phase transformation measurements, spectroscopic ellipsometry scans have been collected for PLD grown thin films of Sb_2Te_3, GST225, and Ge-rich GST. The large spectra measurements (300 – 1700 nm) were conducted at multiple angles of incidence. For analyzing the measurement data, a model was built based on the given heterostructure in the sample: i.e., the substrate, the deposited thin film on top, and the roughness layer. For the optical response representations of the as-deposited and crystalline thin films, the Tauc – Lorentz (TL) model was used. Remember that, unlike Sb_2Te_3 and GST225, the Ge-rich GST thin films have a severe phase separation problem. This phase separation, especially for the crystalline Ge-rich GST thin films, might reduce the data fitting accuracy when (incorrectly) assuming a homogeneous phase in the modelling. The roughness layer was modelled by the effective medium approximation, accounting for 50% thin film layer and 50% air. Fig. S4 shows the refractive index and extinction coefficient values extracted from the best fits for the deposited thin films in both the as-deposited and crystalline phases. A clear trend of reduction in n and k values is visible with increasing Ge-content in the samples. The optical constants extracted from the data fitting for Ge-rich GST thin films and the apparent deviation from the prototypical Sb_2Te_3 and GST225 thin films could be an input for future nanophotonic and optoelectronic devices.

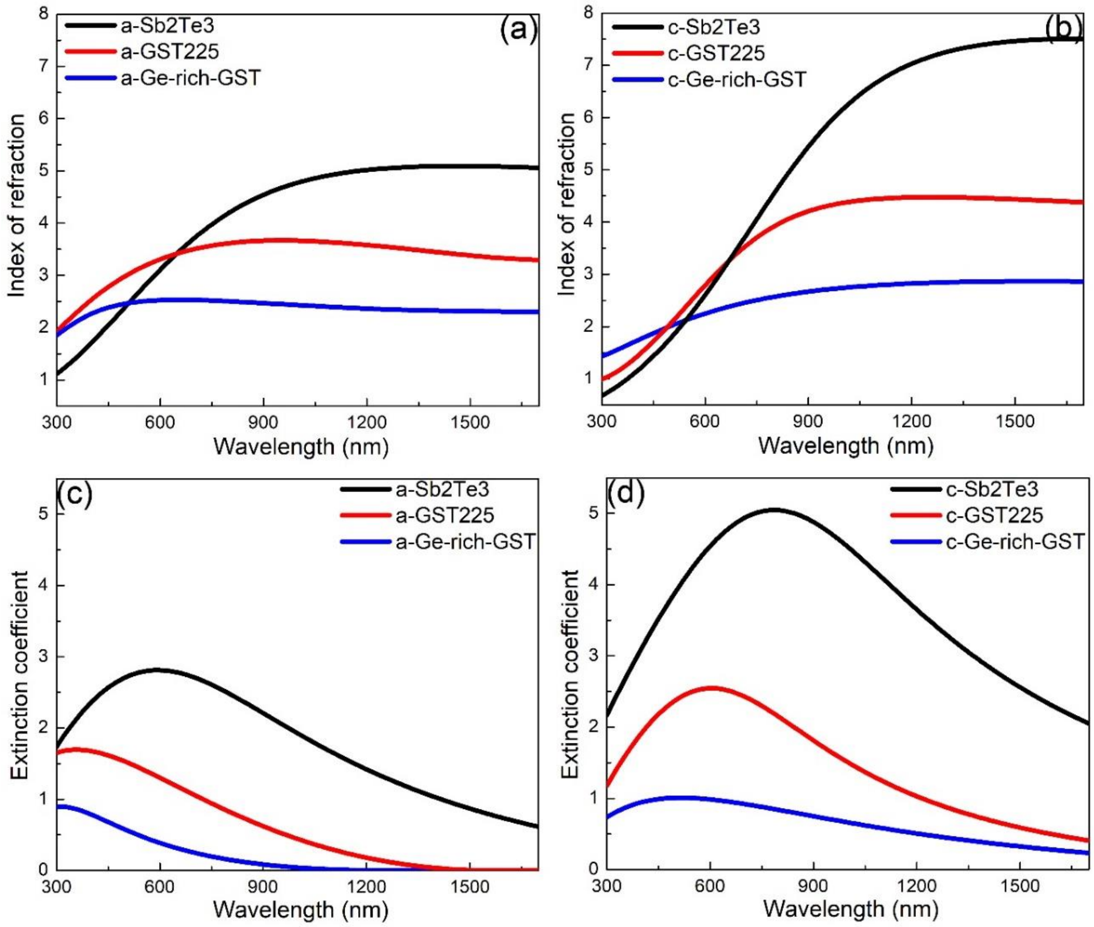

Figure S4. Optical constant data extracted from spectroscopic ellipsometry measurements and data fitting using Tauc–Lorentz optical oscillator. (**a**) Index of refraction for amorphous and (**b**) crystalline samples of Sb_2Te_3, GST225, and GST523 thin films. (**c**) The extinction coefficient for amorphous and (**d**) crystalline samples of Sb_2Te_3, GST225, GST523 thin films.
